# Recurrence of laryngeal squamous cell carcinoma in patients undergoing organ preservation therapy: Are there symptoms associated with recurrence?^☆^^☆☆^^☆☆☆^

**DOI:** 10.1016/j.bjorl.2025.101640

**Published:** 2025-05-27

**Authors:** Sonyara Rauedys Lisboa, Daniel Abreu Rocha, Richard Godoy Mejia, Adolfo Cotarelli Sasaki, Matheus Gerhard Rosenfeld, Leandro Luongo Matos, Daniel Marin Ramos, Marco Aurélio Vamondes Kulcsar

**Affiliations:** a Universidade de São Paulo (USP), Faculdade de Medicina (FM), Hospital das Clínicas (HC), São Paulo, SP, Brazil; b Universidade de São Paulo (USP), Faculdade de Medicina (FM), Instituto do Câncer de São Paulo (ICESP), São Paulo, SP, Brazil

**Keywords:** Laryngeal neoplasms, Larynx, Hypopharyngeal neoplasm

## Abstract

**Objectives:**

This study aims to evaluate whether patients with hypopharyngeal and/or laryngeal Squamous Cell Carcinoma (SCC) who underwent Organ Preservation Therapy (OPT) present at the time of relapse some factor that determines the local recurrence of the disease.

**Methods:**

Patients submitted to OPT were selected at the Cancer Institute of the State of São Paulo (ICESP), at the end of treatment, from January 2012 to December 2017. We collected retrospective data on demographics, clinical staging, location of the primary tumor, presence or absence of recurrence, weight and percentage of weight loss at different moments, alimentary pathway and symptomatology at the time of relapse.

**Results:**

The absence of symptoms was associated with the absence of relapse (*p* < 0.001). Fully oral diet at the last visit was a significant factor for the absence of relapse (*p* = 0.005). The weight comparison of all the patients before the beginning of OPT and after the end of the treatment, showed an average drop of 3.4 kg. In the group-separated analysis, patients who did not recur showed an average loss of 0.7%. Patients with relapse, showed a loss of 2.0% of the weight at the time of relapse.

**Conclusion:**

Weight loss and the presence of symptoms were important predictors of recurrence with statistical significance. These factors may help to better manage these patients, with earlier investigations and, therefore, the possibility of rescue treatments with a shorter duration.

**Level of evidence:**

Level III.

## Highlights

• The absence of symptoms was associated with the absence of relapse (*p* < 0.001), especially pain.
• A wholly oral diet at the last visit was a significant factor in the absence of relapse (*p* = 0.005).
• Weight loss of 2.0% is an essential predictor of recurrence with statistical significance.

## Introduction

The larynx and laryngopharynx are organs of the upper aerodigestive tract that extend from the oropharynx, at the superior border, to the esophagus and trachea, at the inferior. Divided into subunits (supraglottis, glottis and infraglottis), the larynx presents different lymphatic drainage patterns and, together with the laryngopharynx, plays an essential role in protecting the airways during swallowing.^1^ Squamous cell carcinoma is the most common malignant neoplasm affecting these regions. Location in the laryngopharynx is less common, with incidence varying between 5% and 8%.^2^ The larynx is the second most common location of head and neck squamous cell carcinoma.^3^ An increased incidence was observed in both men and women in developing countries, in contrast with the situation in developed countries.^2^ These carcinomas have common risk factors, including excessive tobacco and alcohol consumption.

The laryngopharynx neoplasm presents one of the worst survival rates among head and neck neoplasms, with only 30%‒35% within five years.^4^ Recurrence is common, with around 50% relapse in the first year after diagnosis.^4^

Despite recent advances in chemotherapy, radiotherapy and reconstruction surgery, there is still no preferential treatment for laryngopharyngeal neoplasms.^5^ There is currently, however, a global trend toward therapeutic strategies aiming at organ preservation. Laryngeal neoplasm therapies are based on three main alternatives: total laryngectomy, partial laryngectomies and radiotherapy associated or not with systemic therapy.^6^, ^7^ These options are determined not only by local control of the disease but also by organ functionality.

Considering that patient-related factors, such as nutritional and functional status, are directly related to lower cure rates^8^ and that early diagnosis has an important role in treatment outcome, this study aims at evaluating potential predictors of recurrence of laryngeal and laryngopharyngeal squamous cell carcinoma in patients subjected to Organ Preservation Therapy (OPT), in order to support better follow-up of patients with laryngeal and laryngopharyngeal neoplasms.

## Methods

### Sample

This work aimed to evaluate whether patients with laryngopharyngeal and/or laryngeal squamous cell carcinoma who underwent OPT present, at the time of relapse, any factor that determines local relapse of the disease.

We selected patients submitted to OPT at the Instituto do Câncer do Estado de São Paulo (ICESP) with treatment ending between January 2012 and December 2017. The study has been approved by the institution’s Research Ethics Committee under protocol number 228/14.

The study included retrospective data on demographic characteristics, clinical staging, primary tumor location, presence or absence of relapse, patient’s weight and percentage of weight loss at different times (immediately before the start and after the end of treatment; at relapse or on last medical visit for those who did not relapse; three months before relapse or last visit for those who did not relapse), feeding route and symptomatology at the time of relapse or last visit for those who did not relapse (symptoms evaluated were: new pain complaint or worsening of previous pain pattern; worsening of eating pattern, dyspnea or dysphonia).

The inclusion criterion was that the primary lesion should be squamous cell carcinoma restricted to the larynx and/or laryngopharynx. The exclusion criteria were evolution with local relapse, distant metastasis or second primary tumor; death before, during or immediately after treatment for the primary tumor; loss of follow-up; diagnosis of Disease Persistence (DP) less than three months after completion of primary lesion treatment; presence of primary tumor from another site or distant metastasis at initial presentation; need for total laryngectomy for a cause other than cancer rescue.

Data obtained by the study of each quantitative parametric distribution variable were treated and described using the mean and standard deviation. Absolute and relative frequencies were used for the qualitative analyses. Distributions were defined as parametric by the Kolmogorov-Smirnov test. Student’s *t*-test was used to compare the means of two independent parametric sample populations. Chi-square or Fisher’s exact tests were used to compare frequencies between groups. We used the SPSS 24.0 statistical software (SPSS Inc; Illinois, USA) in all analyses, and adopted a level of statistical significance below 5% (*p* ≤ 0.05) for all comparisons.

## Results

**Fig. 1:**
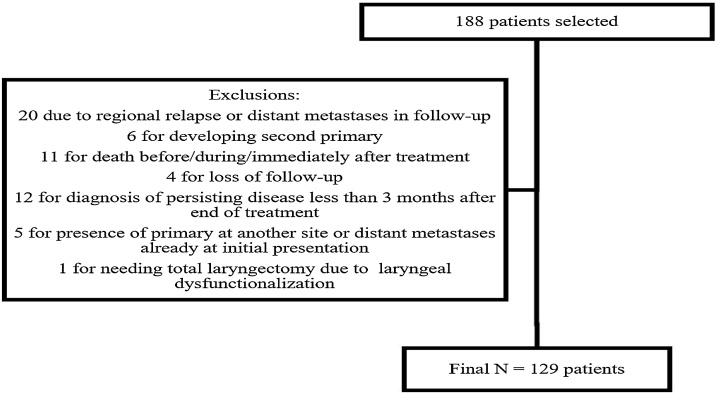
CONSORT diagram showing the selection of patients for the study.

Initially, 188 patients were selected, as shown in Fig. 1.

The average age of patients at the start of treatment for the primary tumor was 61.3 years of age, ranging from 40 to 87 years.

**Fig. 2:**
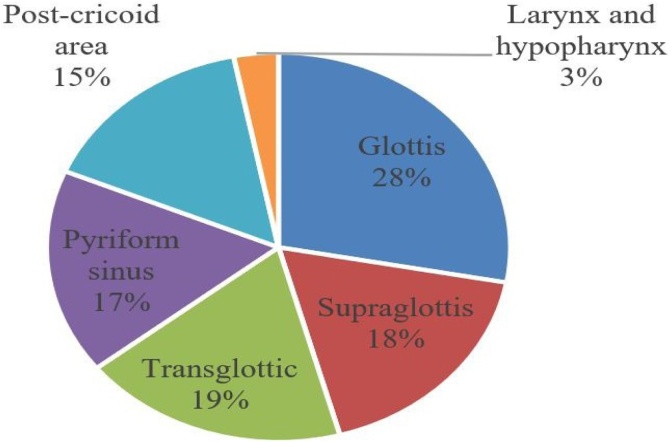
Distribution of cases according to primary tumor topography.

As for the location of the primary tumor, most patients had lesion in the glottis (36 cases), followed by transglottic tumors (Fig. 2).

Patients were clinically classified according to the eighth edition of TNM, aided by direct laryngoscopy performed at the doctor’s office and radiological examination by computed tomography or magnetic resonance imaging (Table 1).

**Table 1:** Distribution of all cases by T and N clinical stage classifications.

Tis	0.8%	N0	49.61%
T1a	8.7%	N1	11.63%
T1b	7.9%	N2	24.81%
T2	13.5%	N3	13.95%
T3	24.6%		
T4a	23.0%		
T4b	21.4%		

Forty-nine of the patients evaluated (38%) presented relapse or local DP, and 80 (62%) did not relapse during follow-up. The mean time for relapse/DP was 1-year.

Most patients with no relapse presented the primary tumor in the glottis (36.3%), predominantly with cT3 lesions; as for patients who relapsed, the majority presented transglottic lesions (22.4%) at stage cT4 (Table 2, Table 3).

**Table 2:** Initial topography of primary tumor for the groups without relapse and with relapse.

	Glottis	Supraglottic	Transglottic	Pyriform sinus	Post-cricoid area	Larynx and laryngopharynx
Without relapse	36.3%	16.3%	16.3%	15%	13.8%	2.5%
With relapse	16.3%	20.4%	22.4%	20.4%	18.4%	4.1%

**Table 3:** Initial T clinical staging of cases divided per group without relapse and with relapse.

	Tis	T1	T1b	T2	T3	T4a	T4b
Without relapse	1.3%	13%	10.4%	14.3%	30%	18%	13%
With relapse	0%	2%	4.1%	12.2%	16.4%	30.6%	34.7%

Regarding the group with no relapse, 66 patients (81.3%) had no symptoms at the last consultation, whereas 14 patients (17.5%) presented symptoms. Of the group with relapse/DP, eight patients (16.3%) did not present symptoms at diagnosis, whereas 41 patients (83.7%) presented symptoms. Absence of symptoms was associated with the absence of relapse (*p* < 0.001 – Chi-Square test) in this group of patients. The group of patients with symptoms was subdivided into five categories (Table 4).

**Table 4:** Distribution of patients with symptoms in absolute numbers divided per group without relapse and with relapse.

	New pain complaint/worsening of previous pain level	Worsening of nutritional pattern	Worsening of dyspnea	Worsening of dysphonia	Mixed (two or more symptoms)
Without relapse	3	4	2	3	1
With relapse	22	8	3	1	7

The patients’ feeding route was assessed at the last consultation and classified as total oral nutrition, mixed or total enteral nutrition. In the comparison between patients without relapse and those with relapse/DP, oral nutrition was the main modality (81.3% vs. 55.1%), followed by total enteral nutrition (16.3% vs. 34.7%) and mixed (2.5% vs. 10.2%). The presence of total oral nutrition at the last consultation was a significantly important factor for the absence of relapse (*p* = 0.005 – Chi-Square test).

Weight comparison of all patients before starting organ preservation treatment and after the end of treatment showed an average drop of 3.4 kg (initial mean weight of 62.5 kg against final mean weight of 59.1 kg). Sixty-seven percent of patients who presented relapse or DP lost weight after treatment; however, this condition was even more frequent in patients who were free of disease during follow-up (83.8%) (*p* = 0.030 – Chi-Square test).

For the analysis of weight comparison at the time of relapse/last consultation with the consultation three months earlier, patients with total enteral diet were excluded, as this is a potential misleading variable in weight maintenance (Table 5).

**Table 5:** Comparison between weight and weight loss and relapse or local Disease Progression (DP) in outpatient assessments.

Variable	Absence of relapse/DP^a^	Presence of relapse/DP^a^	p-value^b^
Weight immediately before start of treatment	65.8 ± 15.7 kg	58.9 ± 11.2	0.201
Weight immediately after start of treatment	62.0 ± 13.9 kg	57.7 ± 11.7	0.680
Percentage of weight loss after treatment	−5.0 ± −8.0%	−1.4 ± −13.3%	0.214
Weight three months before last consultation for those without relapse or three months before relapse for those who	63.5 ± 14.5 kg	58.7 ± 11.2 kg	0.554
Percentage of weight loss three months before (considering relapsed weight after treatment)	2.6 ± 10.7%	2.3 ± 9.1%	0.187
Weight at the last medical consultation for those without relapse or at the moment of relapse for those who relapsed	63.3 ± 14.5 kg	57.0 ± 9.4 kg	0.210
Percentage of weight loss at relapse or at last consultation (considering weight after treatment)	2.2 ± 11.2%	0.1 ± 11.8%	0.930
Percentage of weight loss at relapse or at last consultation (considering weight 3-months before)	−0.7 ± 4.1%	−2.06 ± 9.1%	0.005

Note: “weight loss” with negative averages mean weight loss, and with positive averages, weight gain at observations.

a Mean ± standard deviation.

b T-paired test.

In the analysis per group, the weight of patients who did not relapse at the last consultation was 63.4 kg, while in their visit three months before the average was 63.7 kg, which represents a mean loss of 0.7%. As for those patients who presented relapse/DP, the mean weight at the time of relapse was of 55.8 kg, while in their consultation three months before diagnosis, the average was 57.5 kg ‒ a loss of 2.0% of weight at the moment of relapse. The percentage of weight loss was significantly higher (2.1%) at relapse as compared with their weight three months before diagnosis (p = 0.005 – Student’s *t*-test).

## Discussion

Laryngopharynx Squamous Cell Carcinoma (SCC) presents one of the worst prognoses among head and neck neoplasms, although it has a better prognosis when located in the larynx, where it shows greater incidence. However, both present higher rates of diagnosis at advanced stages, which may reflect the scarcity of symptoms (in cases of laryngopharynx and sub/supraglot) as well as the difficulty of access to specialized health services. A retrospective study undertaken at a major Brazilian center in 2013 revealed a diagnostic rate of up to 94.7%^9^ in cases of laryngopharyngeal squamous cell carcinoma stage III‒IV. Six years later, the present study ratifies these findings showing a higher frequency of cases at the T3-T4a stages.

The treatment of larynx/laryngopharynx neoplasms rests on three pillars: surgery, chemotherapy and radiotherapy. The current trend in managing malignant larynx neoplasms is surgical treatment, particularly partial laryngectomies, for better local control of the disease and to reduce relapse rate. Although the last decades have seen great advancements, laryngopharynx still presents the higher rate of relapse and second primary among head and neck malignant neoplasms.^1^ Bradley et al.:^10^ 47–53 showed that delay over 14 months in beginning treatment corresponded consistently to a higher risk of death.^10^ In the case of patients undergoing post-treatment follow-up (either surgical or chemotherapy/radiotherapy), evaluation and differentiation between recurrence, scarring, edema and local flaps by imaging can be challenging.^11^

Several recent studies have shown that most recurrences of head and neck malignant neoplasms are detected through symptoms reported by patients, instead of physical examination of asymptomatic patients during follow-up.^12^ Boysen et al.:^12^ 1–7 found “[…] a higher rate of symptomatic versus asymptomatic recurrences in glottic and supraglottic recurrences, particularly in the first two years of follow-up”. This finding corroborates with our data, indicating that over 80% of patients with relapses had some complaint, and most asymptomatic patients were non-relapsed. This shows the importance of the patient being able to report new symptoms or changes in previous symptoms, and of the capacity of the medical team in alerting to the prospect of recurrence, aiming at early diagnosis to improve treatment and survival.

Weight loss is an important marker among patients with laryngeal and laryngopharyngeal squamous cell carcinoma, and an important criterion in choosing the treatment to be adopted. We showed that weight loss during post-treatment follow-up is also an important predictor of relapse, as patients without recurrence kept their weight practically constant during/between consultations, and those with confirmed recurrence presented a mean weight loss of 2% in the three months before diagnosis. The presence of symptoms should also serve as a warning during follow-up. We found a symptomatology rate above 80% among relapsed patients, while absence of symptoms prevailed among those with no recurrence. Thus, we have shown that both weight loss and the presence of symptoms during follow-up are extremely relevant factors in post-treatment care of these patients, as they signal the possibility of relapse of the squamous cell carcinoma and the need for earlier diagnostic investigation.

We know that guaranteeing a suitable feeding route ‒ whether oral, enteral or via gastrostomy ‒ for laryngeal and laryngopharyngeal squamous cell carcinoma patients is crucial for their care both pre- and post-treatment. However, data from the available literature recommend a “wait and see” approach regarding nutritional support, and so far, benefits in introducing enteral diets or via gastrostomy prophylactically have not been shown.^13^ It is up to the multidisciplinary medical team (surgeons, nutrologists, nutritionists and speech therapists) to decide on the introduction of complementary feeding routes, taking into consideration factors such as weight loss, incapacity for suitable oral feeding, risk of bronchoaspiration and those inherent to the passage of gastric tubes. In this study, we observed that total oral feeding was statistically associated with the absence of relapse, which is closely related to the natural progression of the disease in sites directly linked to the patient’s swallowing capacity.

The present study aims at finding predictive factors for relapse of laryngeal and laryngopharyngeal squamous cell carcinoma after organ preservation therapy, in view of the technical and economic/administrative difficulties imposed to follow-up of these patients in a major referral center. Taking into account the small sample used in the study and the fact that weight loss, although relevant, proved to be small, we should use the data presented in a relative manner, adopting a case-by-case analysis to find the best course of action according to the peculiarities of each patient. However, as weight loss and the presence of symptoms have proven to be important and statistically relevant predictors of relapse, we believe we have contributed to the improvement of these patients’ management by means of earlier investigations, thus allowing for rescue treatments within a shorter period of time.

## Financial support

None.

## Declaration of competing interest

The authors declare no conflicts of interest.
